# Genomic style: yet another deep-learning approach to characterize bacterial genome sequences

**DOI:** 10.1093/bioadv/vbab039

**Published:** 2021-12-01

**Authors:** Yuka Yoshimura, Akifumi Hamada, Yohann Augey, Manato Akiyama, Yasubumi Sakakibara

**Affiliations:** Department of Biosciences and Informatics, Keio University, Yokohama 223-8522, Japan

## Abstract

**Motivation:**

Biological sequence classification is the most fundamental task in bioinformatics analysis. For example, in metagenome analysis, binning is a typical type of DNA sequence classification. In order to classify sequences, it is necessary to define sequence features. The *k*-mer frequency, base composition and alignment-based metrics are commonly used. On the other hand, in the field of image recognition using machine learning, image classification is broadly divided into those based on shape and those based on style. A style matrix was introduced as a method of expressing the style of an image (e.g. color usage and texture).

**Results:**

We propose a novel sequence feature, called genomic style, inspired by image classification approaches, for classifying and clustering DNA sequences. As with the style of images, the DNA sequence is considered to have a genomic style unique to the bacterial species, and the style matrix concept is applied to the DNA sequence. Our main aim is to introduce the genomics style as yet another basic sequence feature for metagenome binning problem in replace of the most commonly used sequence feature *k*-mer frequency. Performance evaluations showed that our method using a style matrix has the potential for accurate binning when compared with state-of-the-art binning tools based on *k*-mer frequency.

**Availability and implementation:**

The source code for the implementation of this genomic style method, along with the dataset for the performance evaluation, is available from https://github.com/friendflower94/binning-style.

**Supplementary information:**

Supplementary data are available at *Bioinformatics Advances* online.

## 1 Introduction

Classification (or clustering) of biological sequences (DNA, RNA and protein sequence) is among of the most fundamental tasks in bioinformatics analysis. In order to classify sequences, the sequence features must be defined, for which three main methods are most frequently used: homology, *k*-mer (word of length *k*) frequency and base composition [e.g. guanine-cytosine (GC) content]. In the first method, homology is scored by performing alignment and calculating the score of the matched positions and the mismatched positions, and the resulting score is then used for classification (Durbin *et al.*, 1998). The *k*-mer frequency, in the second method, is the frequency of occurrence of a sub-sequence (word) of length *k*. A profile is obtained from this frequency distribution, which is expected to be specific to bacterial species, and classification is performed by comparing the similarities of profiles. In the third method, the base composition, particularly, the GC content, is the percentage of GC among the four bases in the sequence. *Streptomyces coelicolor* is an actinomycetes species considered to have a high GC content (72.2%) that greatly differs from that in *Escherichia coli* (50.6%). The third method uses this difference in the GC content to classify sequences. Sequences can be classified using these values that characterize DNA sequences. However, using these methods, the classification accuracy is not always high.

An example of the application of DNA sequence classification is binning, which classifies the obtained DNA sequences to each species in metagenomic analysis (Mande *et al.*, 2012). Metagenome analysis is a method for comprehensively analyzing the DNA extracted from an environment in which multiple species are mixed. Because the intestinal flora is known to affect human health and disease, metagenome analysis is attracting attention as a research method to clarify which bacteria are present in the intestinal flora and how they interact with the host. In metagenomic analysis, the total DNA of a mixed sample of various bacteria is extracted and sequenced. The reads obtained by sequencing are subjected to processes, such as assembly to reconstruct the genome and binning to classify the sequences according to the bacterial species. As the sample contains many unknown bacteria, the genomes of which have not been determined, it is important to perform the binning processing to classify the sequences to each species.

Most existing binning methods have been developed to bin contigs assembled from sequence reads, and are categorized into supervised (classification) and unsupervised (clustering) approaches. These methods are further classified into alignment-based and alignment-free ones. The supervised approaches including MEGAN (Huson *et al.*, 2007) and Kraken (Wood and Salzberg, 2014) require the reference database or training data with the taxonomic class labels in order to assign assembled contigs to correct taxonomic classes. In comparison, the unsupervised approaches do not require this information. In metagenomic analysis, as many bacteria species in the bacterial flora are unknown and recent next-generation sequencing technique generates a large amount of sequence data, the unsupervised and alignment-free methods are more demanded. In the alignment-free method, most binning methods make use of the *k*-mer frequency (*k*-mer composition) as the fundamental sequence feature, such as the tetra-mer composition (Chatterji *et al.*, 2008; Yang *et al.*, 2010). In addition, the accuracy can be improved by incorporating the coverage information that represents the species abundance in the bacterial flora [MetaBAT2 (Kang *et al.*, 2019), CONCOCT (Alneberg *et al.*, 2014), MaxBin2 (Wu *et al.*, 2016), MetaProb (Girotto *et al*, 2016) and MrGBP (Kouchaki *et al*, 2019)]. However, the accuracy of binning that relies on the *k*-mer frequency is not sufficient. In this study, we propose the ‘genomic style’ inspired by deep-learning for image classification as yet another new sequence feature that replaces the *k*-mer frequency basically used in existing binning methods.

In the field of deep learning-based image processing (Jing *et al.*, 2020; Kang, 2015), image classification is largely divided into those based on shape and those based on style. The former is a classification based on the shape of what is drawn in the image, and the latter is a classification based on artistic style of an image, such as color usage and texture, which is unique to the creator of the image. As the artistic style of an image is a rich descriptor that captures both visual and historical information about the painting, several studies have addressed the problem of style recognition by employing the machine-learning technique to automatically detect the artistic style of paintings (Lecoutre *et al.*, 2017).

Recently, a deep-learning approach known as neural style transfer (Gatys *et al.*, 2015) has been reported to convert an image to another style while maintaining the image content (shape and arrangement of objects). The concept of style matrix was introduced to express the artistic style. The style matrix consists of correlation values between the feature maps of different filter responses in the same hidden layer of the convolutional neural network (CNN). This definition of style matrix has been hypothesized to represent the style of the image, such as color usage and texture. A distinctive feature of the style matrix is that the style transfer task needs to transfer the style to a new image while preserving the content of the image, thus separating the style from the content as much as possible. This feature is suitable for our purpose to extract the style of a genome sequence specific to a bacterial species while preserving the content of genetic information.

In this study, we hypothesized that the DNA sequence has a style, named as the ‘genomic style’, which is similar to the style of an image and is unique to a bacterial species. We applied the style matrix concept developed in the neural style transfer study to DNA sequences and aimed to extract genomic styles. Then, we proposed a method for clustering of the metagenomic sequences using the extracted genomic style as a sequence feature. We expect that the genomic style opens up new insights into the biological sequence analysis in metagenome analysis and bacterial taxonomy analysis.

## 2 Methods

The outline of the method is as follows. First, we constructed a CNN, called the feature-extraction CNN model, for modeling the content of DNA sequences and extracting sequence features. In order to extract the most general sequence features possible, we trained the feature-extraction CNN model using a wide variety of known bacterial species genomes from 139 different taxa represented at the species level. This corresponds to pre-training to extract the genomic style, defined by the correlation values between extracted feature maps in the feature-extraction CNN model. In this pre-training process, sequence motifs were extracted within the filters of the feature-extraction CNN model as important for taxonomic classification. Second, we calculated the style matrix by inputting a DNA sequence into the trained feature-extraction CNN model and extracted the genomic style of the DNA sequence. Third, we performed binning by clustering DNA sequences using the obtained genomic style as a feature vector. The source code for implementation of this genomic style method, along with the dataset used for the performance evaluation, is available from https://github.com/friendflower94/binning-style.

### 2.1 Style of image

First, we briefly introduce Neural Style Transfer (Gatys *et al.*, 2015), a deep learning-based image-style transfer method that was recently proposed for generating an image converted to another style while maintaining the content of the image. An image whose object layout is to be preserved is called a content image, and an image with a style used for conversion is called a style image. The style, such as color and texture, of the content image is rewritten while maintaining the position and outline of the object in the content image. To accomplish the style transfer task for an image, neural style transfer proposed a method, called ‘style matrix’ to express the style of an image by making use of Visual Geometry Group (VGG) (Simonyan and Zisserman, 2014), a CNN specifically designed for image recognition.

### 2.2 Convolutional and pooling layer in CNN

The CNN, which has demonstrated an epoch-making performance in image analysis, has a convolution layer consisting of filters that automatically learns the features of images, such as lines and circles present in the image. By combining this convolutional mechanism in multiple layers, deeper hierarchies acquire higher-order features, such as facial contours. Another technical feature of CNN is that one image is learned to be decomposed into multiple components known as *feature maps*.

More precisely, in the first convolutional layer, the inner product between the filter of arbitrary size and each local region of the input image is calculated. The filter scans the entire input image with a fixed stride. This process extracts the feature map of the input image. In the deeper hidden layer, the inner product between the filter and the feature map of the previous layer is calculated. Each hidden layer has multiple filters so that the hidden layer outputs the set of feature maps (output one feature map per one filter). The pooling layer calculates the maximum value or average value within a certain range and makes it the representative value within the range. This enables acquiring robustness against the position movement and compressing information in the input image. Additionally, the input to the convolutional layer may include multiple input vectors, known as channels (which are also considered as feature maps in the input layer). For example, when the input to the CNN is an RGB color image, there are three channels: red (R), green (G) and blue (B).

In this study, the input of DNA sequence is encoded to a one-hot coding representation (one-hot vector) of four DNA nucleotides with a height of four dimensions and width of the DNA sequence length. When one-hot coding representation of DNA sequence is employed, then a filter with a one-dimensional convolution operation is applied temporally over a sequence. Here, a ‘one-dimensional’ convolution operation for sequences is interpreted as scanning the input sequence only in one direction along the sequence. Thus, in the following formulas, both the filter and feature map become one-dimensional.

For a filter function in the *l*-th hidden layer of the CNN, the input is the set of feature maps in the (*l*−1)-th hidden layer $v_{i:i+d-1,j}^{\left(l-1\right)}=c_{i,j}^{\left(l-1\right)}\in R^{m\times n}$, where *d* is the filter size, *m* is the size of feature map and *n* is the number of feature maps. The output for the *k*-th filter is a feature map of the *l*-th layer $c_{i}^{\left(l,k\right)}\in R^{m}$, which is defined as follows:

cil,k=fWl,k·ci,jl-1+bl,k,(1)

where *f* is an activation function, $W^{\left(l,k\right)}\in R^{m\times n\times d}$ is the weight matrix of the *k*-th filter in the *l*-th convolutional layer and $b^{\left(l,k\right)}$ is the bias vector.

### 2.3 Style matrix for image style

Neural style transfer (Gatys *et al.*, 2015) proposed a style matrix as a method to represent the artistic style of an image. The style matrix consists of the correlation values between the multiple feature maps of different filter responses in the same hidden layer in CNN (also defined as the Gram matrix of feature maps). In their style transfer experiment to convert an image to another style while maintaining the content of the image, it was revealed that content information, such as the shape and spatial position of the objects in the image can be separated from style information, such as color and texture by using the style matrix.

Neural style transfer uses VGG (Simonyan and Zisserman, 2014) as an image recognition model. VGG16 is a CNN model composed of 13 convolutional layers, 5 pooling layers and 3 fully connected layers. VGG earned the second place in 2014 at ILSVRC, an image recognition competition. VGG is a model that has been trained by ImageNet (Krizhevsky *et al.*, 2012; Lecoutre *et al.* 2017), a dataset containing over 14 million RGB images.

### 2.4 Genomic style

In this study, by applying the style matrix method of style extraction to genome sequences, we extract genomic styles specific to bacterial species, such as the base composition and codon usage.

**Fig. 1.**
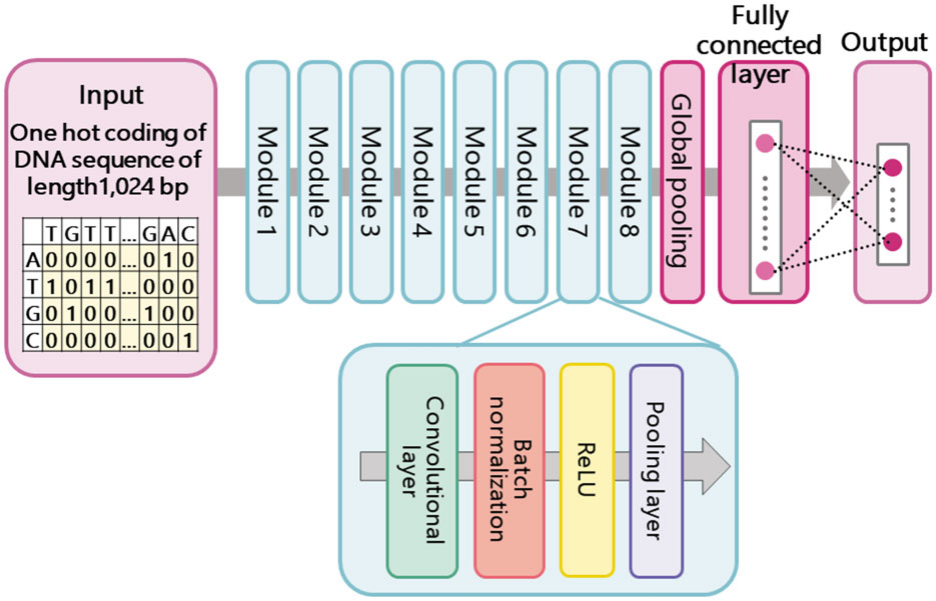
Feature-extraction CNN model for modeling the content of DNA sequences. The feature-extraction CNN model is trained for the task of bacterial species classification

First, we constructed the feature-extraction CNN model for modeling the content of DNA sequences by performing bacterial species classification learning. The species classification learning with a wide variety of known bacterial species genomes was expected to extract the most general features possible. This feature-extraction model is a CNN model in which eight modules consisting of the convolutional layer, batch normalization, rectified linear unit and the pooling layer are stacked, and finally, the output is obtained through the global average pooling layer and the fully connected layer (displayed in Fig. 1). The cross-entropy error function for multi-class classification was used as the loss function to be minimized in learning.

The input is a one-hot coding representation (one-hot vector) of four DNA nucleotides with a height of four dimensions and a width of 1024 dimensions as the maximum length of input DNA sequences. Several previous studies (Alipanahi *et al.*, 2015; Aoki and Sakakibara, 2018; Kelley *et al.*, 2016; Zeng *et al.*, 2016; Zhou and Troyanskaya, 2015) showed that a CNN can be applied to extraction of a sequence motif specifically conserved among target DNA sequences. When one-hot coding representation of four DNA nucleotides is employed, then a filter with a one-dimensional convolution operation applied temporally over a sequence can be considered as a position weight matrix for representing a motif.

**Fig. 2.**
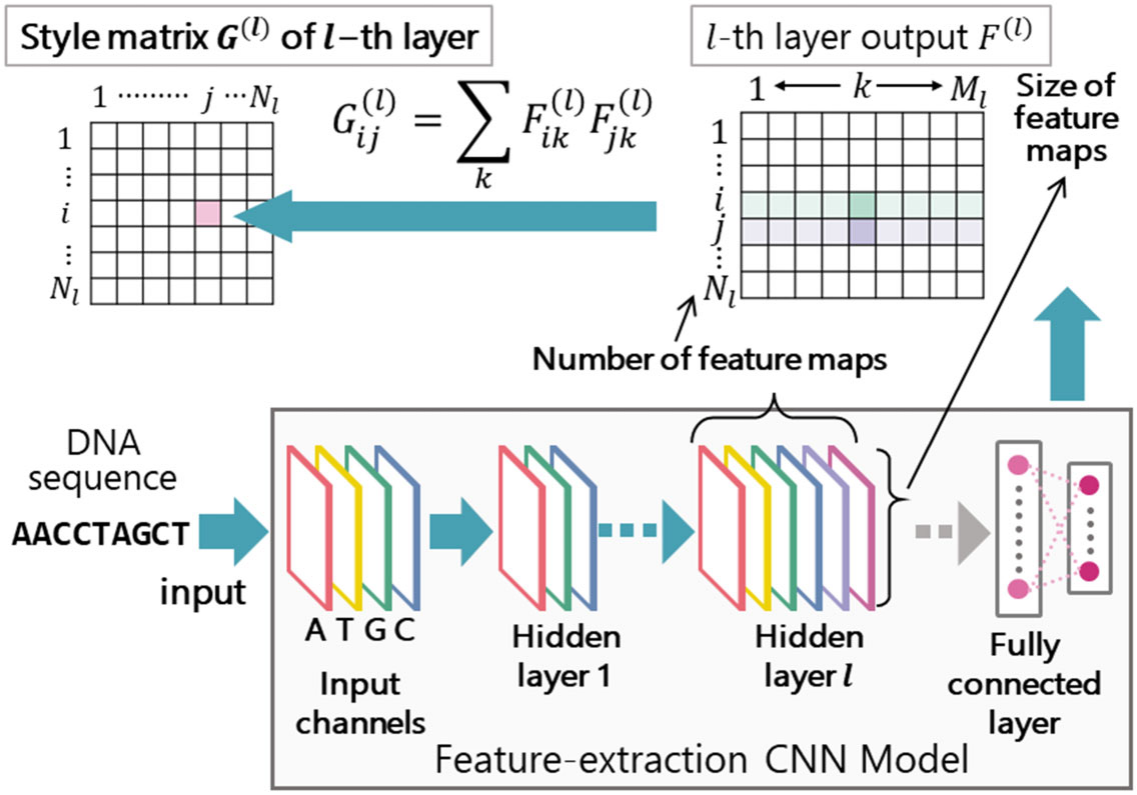
Calculation of style matrix $G_{ij}^{\left(l\right)}$ of the *l*-th hidden layer. The style matrix of the DNA sequence, called *genomic style*, is calculated using the feature-extraction CNN model for the input DNA sequence

Second, the style matrix of the test DNA sequence, called *genomic style*, was calculated using the feature-extraction CNN model with the trained parameters. In the *l*-th hidden layer, when $N_{l}$ denotes the number of feature maps and $M_{l}$ denotes the size of the feature map, the *l*-th layer output $F^{\left(l\right)}$ becomes $F^{\left(l\right)}\in R^{N_{l}\times M_{l}}$. Let $F_{ij}^{\left(l\right)}$ denote the *j*-th element of the *i*-th feature map in the *l*-th layer. Then, the element $G_{ij}^{\left(l\right)}$ of the style matrix $G^{\left(l\right)}$ of the *l*-th layer is defined as the inner product between the *i*-th feature map and the *j*-th feature map in the *l*-th layer, as shown in the following formula and displayed in Figure 2:

Gijl=∑kFikl·Fjkl,#2

By inputting the test DNA sequence into the feature-extraction CNN model, the style matrix of the input test DNA sequence is calculated using the above formula. We use this style matrix as the feature vector for clustering the test DNA sequences, where the feature vector is constructed by listing the rows of the style matrix in order.

### 2.5 Binning using genomic style

Binning is the process of clustering DNA sequences from a mixed sample of multiple species into bins of the same species (Thomas *et al.*, 2012). We perform binning by clustering DNA sequences using the obtained style matrix as a feature vector. Because the style matrix $G^{\left(l\right)}$ can be obtained for each layer, the style matrix is calculated for the first to sixth layers, i.e. *l* = 1, 2, 3, 4, 5 and 6, and binning is performed using each layer in the following experiment.

The input to the feature-extraction CNN model has a fixed sequence length of 1024 bp. As the length of the DNA sequence to be binned is longer than 1024 bp, the input DNA sequence is scanned with a fixed length of 1024 bp and shifted by 500 bp, and the style matrix for each 1024 bp is calculated. The average of these values is used as the style matrix for the entire input sequence. The averaging operation for the style matrices is equal to calculating the frequency of style matrices, and therefore is a straightforward extension of the *k*-mer frequency and similar to the average pooling operation in the CNN model.

We employed the agglomerative clustering method (Frigui and Krishnapuram, 1997) for clustering with the style matrix. This method starts with a cluster consisting of one element and recursively merges two clusters with the minimum inter-cluster distance until the target number of clusters is reached. The Euclidean distance of the style matrix was used for the distance between elements, and the distance between clusters is calculated using the Ward method.

In addition, for comparison with state-of-the-art binning tools, we incorporated the coverage (abundance) information into binning using the style matrix. The extension simply added another dimension [(*L* + 1)-th dimension] into the (*L*-dimensional) feature vector to represent the coverage value of each DNA sequence, where the coverage value of DNA sequence is the average of coverages of all positions in the DNA sequence. We adjusted the scale of the coverage value to fit the scale of the style matrix. Agglomerative clustering with Euclidean distance was applied to the extended feature vector. We named this extension as the ‘style matrix with coverage’.

### 2.6 Existing binning methods for performance comparison

The purpose of our experiment is to compare the ability of the proposed sequence feature, genomic style in replace of *k*-mer frequency for the binning task.

As the baseline for performance comparison, binning was performed using the ‘*k*-mers frequency’. For each DNA sequence, the number of occurrences of sub-sequences of length *k* was counted by scanning and shifting by one base. The number of occurrences was normalized by dividing by the total number to obtain the *k*-mers frequency. The *k*-mers frequency was calculated for *k* = 3 and 4, and clustering was performed using agglomerative clustering with *k*-mers frequency as the feature vector.

To compare the performance with state-of-the-art binning tools, the following tools were applied in our second experiment: MetaBAT2 (Kang *et al.*, 2019), CONCOCT (Alneberg *et al.*, 2014), MaxBin2 (Wu *et al.*, 2016) and MrGBP (Kouchaki *et al.*, 2019). Default values were used for all parameters in these methods. MetaBAT (Kang *et al.*, 2015) is an automated binning software that can process large datasets of DNA sequences and is based on empirical probabilistic distances of tumor necrosis factor (TNF), i.e. the 4-mers frequency and genome abundance. MetaBAT is widely used by the microbiology community; a new version of MetaBAT has been released named as MetaBAT2 (Kang *et al.*, 2019), which is a state-of-the-art binning tool that automates tuning of the parameter to obtain better binning results. MetaProb (Girotto *et al.*, 2016) failed to accomplish the binning experiment for the Critical Assessment of Metagenome Interpretation (CAMI) challenge dataset; therefore, we removed this tool from our experiment. All existing binning tools utilize coverage (abundance) information for each sequence as well as the *k*-mers frequency.

## 3 Datasets

The experiment proceeded with four different datasets. The first dataset was used for pre-training of the feature-extraction CNN model. The second dataset consisted of assembled contigs to compare the effectiveness of genomic style and *k*-mer frequency as sequence features for the binning task, and the third dataset was obtained from the CAMI challenge dataset (Sczyrba *et al.*, 2017) for the performance comparison with the existing binning methods. The fourth dataset was comprised of random DNA sequences with different GC contents.

### 3.1 Training dataset

As the training set, DNA sequences of bacterial whole genomes obtained from the National Center for Biotechnology Information Nucleotide database (Sayers *et al.*, 2019) were used. We collected a wide variety of known bacterial genomes from 206 different taxa represented at the species level. The list of these 206 bacterial species and their taxonomic hierarchy are presented in Supplementary Table S1 and Figures S1 and S2. The dataset was divided into 139 species used as training data to train the model and 67 species for the binning test data without duplication. To construct the dataset for pre-training of the feature-extraction CNN model, we used 139 whole genomes, each one belonging to different species. Next, DNA sequences of 1024 bp in length were randomly extracted from each genome. As a result, we constructed 128 000 DNA sequences as the training dataset.

### 3.2 Binning test dataset to compare genomic style and *k*-mer frequency

A total of 67 species were used for the binning test data to compare the genomic style and the *k*-mer frequency. To produce the binning test dataset for assembled contigs, we used CAMISIM software (Fritz *et al.*, 2019) to simulate the metagenomes. We used the ART read simulator to generate reads from a set of pre-specified genomes. It produced reads simulated from the Illumina HiSeq150 sequencing technology. The fragment size mean was set to 270 bp, and the fragment standard deviation was set to 27 bp. We used the golden standard assembly generated by CAMISIM, i.e. the set of contigs with their associated ground-truth labels. We retained contigs with a length of over 10 000 bp and cropped the larger ones to obtain contigs of length 10 000 bp. We performed this step seven times to obtain a binning test dataset consisting of 57 818 contigs, each with a length of 10 000 bp and originating from 67 different species.

**Fig. 3.**
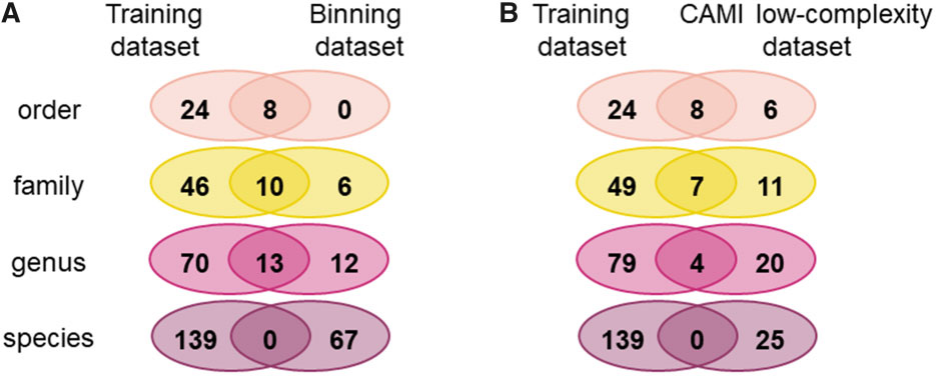
Common and different taxa between the training dataset and the binning test dataset and between the training dataset and the CAMI low-complexity dataset. For each taxonomic rank, the number of taxa, which are exclusive to the training dataset, which are exclusive to the binning test dataset (**A**), which are exclusive to the CAMI low-complexity dataset (**B**) and which are common to both are shown. Note that in the CAMI low-complexity dataset, the 40 genomes and 20 circular elements included those that were unidentified and those identified up to higher taxonomy in each taxonomic rank

In addition, binning test datasets were created at the genus and family levels, respectively. In the genus-level test dataset, one species from each of the 25 genera was selected, and DNA sequences in the selected species were labeled as that genus. In the family-level test dataset, one species from each of the 16 families was selected, and DNA sequences in the selected species were labeled as that family. In these two datasets, the binning task is expected to be simpler and its classification accuracy will be higher. Figure 3 (left) shows, for each taxonomic rank, the number of taxa which are exclusive to the training dataset, which are exclusive to the binning test dataset and which are common to both.

### 3.3 CAMI challenge dataset

The CAMI challenge (Sczyrba *et al.*, 2017) is an effort to provide gold-standard benchmark datasets for comparing tools for the analysis of metagenomic data analysis. Reads in the low-complexity dataset of the CAMI challenge are simulated with Illumina HiSeq error profiles from 40 genomes and 20 circular elements. The gold-standard metagenome assembly constructed from reads in the CAMI low-complexity dataset contains 19 499 DNA sequences (a set of contigs with their associated ground-truth labels), which are mixtures of variable-length fragments originating from individual species. This DNA sequence dataset is provided as the binning challenge for genome binning (unsupervised binning that is clustering) to group sequences into unlabeled bins and taxonomic binning (supervised binning) to group the sequences into bins with a taxonomic label attached. Figure 3 (right) shows, for each taxonomic rank, the number of taxa exclusive to the training dataset, which are exclusive to the CAMI low-complexity dataset, and which are common to both. Note that, the 40 genomes and 20 circular elements in the low-complexity dataset included those that were unidentified and those identified up to higher taxonomy in each taxonomic rank.

### 3.4 Random DNA-sequences with different GC content

Random DNA sequences with different GC contents were generated to characterize the sequence features acquired by the genomic style matrix. A total of 500 random DNA sequences were generated for each of the two groups with different GC contents of *x*% and (100 − *x*)%. For a total of 1000 generated sequences, we calculated the style matrix and performed clustering by agglomerative clustering. The binning performance was evaluated with a GC content of *x* = 53, 55, 57.

## 4 Results

The following measures were used as the performance evaluation criteria: adjusted rand index (ARI), homogeneity, completeness metrics and *F*-measure.

The ARI (Hubert and Arabie, 1985) is a metric derived from the rand index (RI), defined as:

$$\mathrm{RI}=\frac{\mathrm{TP}+\mathrm{TN}}{\mathrm{TP}+\mathrm{FP}+\mathrm{FN}+\mathrm{TN}},$$

$$\mathrm{ARI}=\frac{\mathrm{RI}-E[\mathrm{RI}]}{\max\left(\mathrm{RI}\right)-E[\mathrm{RI}]},$$

where TP denotes the number of the same species DNA sequences in the same cluster, TN denotes the number of different species sequences in different clusters, FP denotes the number of different species sequences in the same cluster and FN denotes the number of same species sequences in the different cluster. In many cases, the number of sequence pairs in different clusters is greater than that of sequence pairs in the same cluster, so the naive clustering to divide all sequences into different clusters will increase the RI value. Therefore, the ARI is obtained by decreasing as a penalty the value of RI when clustering is performed arbitrarily.

Homogeneity is an index that measures the purity of bins without contamination. If the predicted cluster contains only elements from the same species belong, it is defined to be homogeneity. Completeness is a measure of whether elements belonging to the same species are assigned to the same cluster. Generally, the higher the homogeneity, the lower the completeness is, and vice versa. Thus, homogeneity and completeness are trade-off indices. The precise definitions of homogeneity and completeness are provided in the Supplementary Material. The *F*-measure *f* (balanced *F*-score) is a metric defined as:

$$f=\frac{2\times \mathrm{rec}\times \mathrm{pre}}{\mathrm{rec}+\mathrm{pre}},$$

where rec=TP/(TP+FN) and pre=TP/(TP+FP).

### 4.1 Effectiveness of genomic style as sequence features for binning task

Table 1 shows the binning accuracy using the style matrix from the first to sixth layer and the *k*-mer frequency (*k* = 3, 4) for the binning test datasets at the species level, genus level and family level. Among the style matrices using six different layers, the fourth layer gave the highest accuracy in all four accuracy indices in the species and family-level test datasets, except the homogeneity score in the species level. In the genus-level test dataset, the fifth layer showed the highest accuracy in ARI, completeness scores and *F*-measure, but the differences between the fourth and fifth layers were small. When comparing among three test datasets at the species, genus and family levels, a higher taxonomic level showed higher accuracy. In particular, the ARI score using fourth layer reached 0.797 for the family-level test dataset.

**Table 1.** Performance evaluation of the binning methods using the style matrix from the first to sixth layer, and the *k*-mer frequency (*k* =3, 4)

Method	Style matrix (of layer *l*)						*k*-mers frequency	
	*l* =1	*l* =2	*l* =3	*l* =4	*l* =5	*l* =6	*k* =3	*k* =4
Species								
ARI	0.434	0.415	0.461	**0.492**	0.480	0.316	0.372	0.378
Homogeneity	0.707	0.725	**0.756**	0.754	0.713	0.612	0.675	0.691
Completeness	0.673	0.693	0.722	**0.732**	0.719	0.654	0.640	0.657
*F*-measure	0.449	0.431	0.475	**0.506**	0.496	0.339	0.389	0.394
Genus								
ARI	0.493	0.498	0.603	0.609	**0.703**	0.518	0.497	0.481
Homogeneity	0.758	0.781	0.791	**0.792**	0.779	0.717	0.742	0.767
Completeness	0.700	0.725	0.757	0.783	**0.787**	0.713	0.686	0.704
*F*-measure	0.525	0.531	0.632	0.639	**0.727**	0.553	0.530	0.559
Family								
ARI	0.545	0.739	0.723	**0.797**	0.789	0.623	0.566	0.557
Homogeneity	0.792	0.861	0.868	**0.870**	0.834	0.797	0.803	0.817
Completeness	0.729	0.807	0.812	**0.825**	0.812	0.762	0.738	0.730
*F*-measure	0.590	0.766	0.751	**0.819**	0.813	0.662	0.609	0.597

*Note*: Four accuracy indices, ARI, homogeneity, completeness and *F*-measure are shown for the binning test datasets at the species level, genus level and family level. The highest value for each accuracy indices is shown in bold.

Compared with the *k*-mer frequency, at all taxonomic levels, the accuracies using the style matrix were higher for ARI, homogeneity, completeness and *F*-measure.

### 4.2 Performance comparison with existing binning tools

The binning performance of the style matrix with coverage was compared with that of state-of-the-art binning tools using the CAMI challenge dataset. The style matrix with coverage used the fourth layer, which exhibited the highest performance in the binning test without using the coverage score. The result is shown in Table 2.

**Table 2.** Performance comparison with existing binning methods on CAMI challenge dataset

	Style matrix with coverage (*l* =4)	Style matrix (*l* =4)	MetaBAT2	CONCOCT	MaxBin2	MrGBP
ARI	**0.207**	0.160	0.044	0.165	0.146	0.066
Homogeneity	0.646	0.563	**0.979**	0.925	0.894	0.639
Completeness	**0.400**	0.335	0.243	0.282	0.270	0.219
*F*-measure	**0.266**	0.217	0.053	0.195	0.178	0.177
Num. of unclassified	0	0	15 592	11 063	11 182	11 091

*Note*: The unclassified sequence was considered to form bins consisting of single element. The highest value for each accuracy indices is shown in bold.

The style matrix of the fourth layer with coverage achieved a competitive ARI, completeness and *F*-measure score among all binning methods, whereas MetaBAT2, CONCOCT and MaxBin2 showed very high homogeneity. All existing tools excluded more than half of the DNA sequences in the CAMI challenge dataset as unclassified, resulting in low ARI and completeness scores. Comparison of the style matrix of the fourth layer without coverage information revealed that coverage information helped improve the accuracy of the ARI, homogeneity, completeness and *F*-measure scores. This evaluation result showed that our method using a style matrix has the potential for accurate binning when compared with state-of-the-art binning tools based on *k*-mer frequency. On the other hand, although performance evaluation that excludes the unclassified contigs are more appropriate for assessing the applicability to real-world usage, this study aimed to evaluate the potential of the genomic style rather than developing a practical binning system.

### 4.3 Binning DNA-sequences with different GC content

**Fig. 4.**
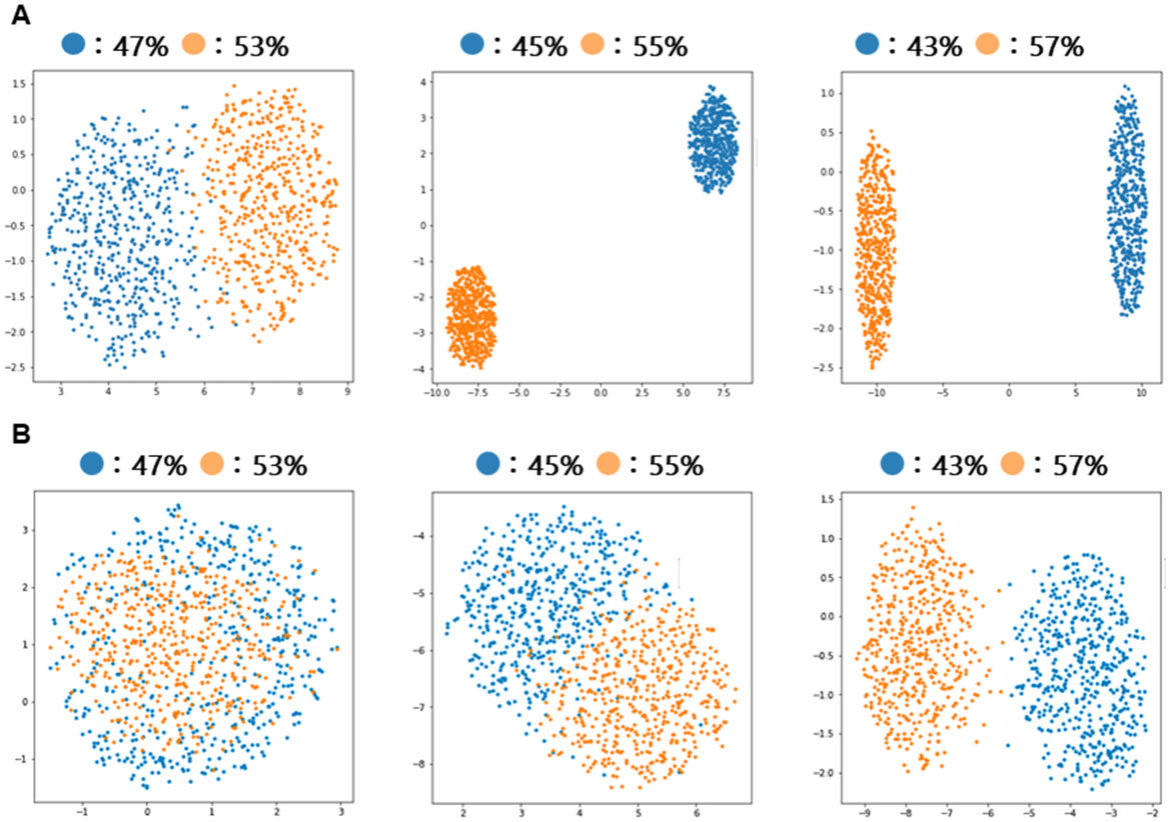
Visualization of the results of clustering DNA sequences with different GC content by reducing the dimension of the style matrix to two dimensions using UMAP. (**A**) Clustering using the style matrix of second layer. (**B**) Clustering using the style matrix of fourth layer

Table 3 shows the result of clustering 1000 randomly generated sequences with different GC contents by using the style matrix of the second and fourth layers. Figure 4 displays the visualization of clustering the DNA sequences by reducing the dimension of the style matrix to two dimensions using Uniform Manifold Approximation and Projection (UMAP). UMAP (McInnes *et al.*, 2018) is a dimension compression tool that performs dimension reduction considering non-linear components at high speed. It turned out that both style matrices of the second and fourth layers could separate sequences with different GC contents, with the style matrix of second layer showing higher accuracy compared to the fourth layer. In the plots displayed in Figure 4, DNA sequences with larger difference in GC contents were more clearly separated, and the style matrix of the second layer separated the two groups with different GC contents more explicitly compared to the style matrix of the fourth layer.

**Table 3.** Binning accuracy using the style matrix of second and fourth for random DNA sequences with different GC content

Method	ARI	Homogeneity	Completeness
Group 1 (GC: 53%), Group 2 (GC: 47%)			
Style matrix of layer 2	0.732	0.627	0.627
Style matrix of layer 4	0.000	0.000	0.000
Group 1 (GC: 55%), Group 2 (GC: 45%)			
Style matrix of layer 2	1.000	1.000	1.000
Style matrix of layer 4	0.611	0.503	0.504
Group 1 (GC: 57%), Group 2 (GC: 43%)			
Style matrix of layer 2	1.000	1.000	1.000
Style matrix of layer 4	0.910	0.847	0.848

## 5 Discussion

The accuracy of binning with the style matrix constructed in this study was highest when the style matrix of the fourth layer was used. In performance comparison with existing binning tools, the style matrix of the fourth layer with coverage achieved higher accuracy in ARI, completeness and *F*-measure compared to state-of-the-art methods. With regard to homogeneity, MetaBAT2, CONCOCT and MaxBin2 showed higher accuracy than the other methods. These methods excluded more than half of the DNA sequences in the CAMI challenge dataset as unclassified. The unclassified sequence was considered to form bins consisting of single element. Homogeneity measures whether each cluster contains only elements from the same species. Obviously, bins containing single element are homogeneous, and the homogeneity value tends to be higher when more bins with a smaller number of elements are generated.

Evaluation of the style matrix for the generated sequence with different GC contents revealed that the style matrix of both the second layer and fourth layers could distinguish the differences in GC contents. Therefore, the style matrix considers differences in GC contents and base composition depending on the bacterial species. In the second layer, the sequence can be separated to some extent even if the GC content difference is 6%, whereas in the fourth layer, the sequence cannot be separated unless the difference exceeds 10% in GC content. Therefore, the GC content was considered to have been captured in the shallow layer.

The feature map itself in the feature-extraction CNN model did not function as a feature for DNA sequence clustering (unsupervised classification). The feature map is the filter responses from the previous layer, i.e. the inner product (convolution operation) between the filter and each local region of the previous layer’s output (this is also the feature map), as defined in the Equation (1). The feature map acquired some features for the content of the input DNA sequence. However, Table 4 shows that the binning accuracies using feature maps of the second, fourth and sixth layers were very low. This result indicates that calculation of the ‘correlation values’ (i.e. the style matrix) between multiple feature maps was necessary as the sequence feature for binning DNA sequences.

**Table 4.** Binning accuracy using the feature maps as sequence features for clustering

Method	ARI	Homogeneity	Completeness
Feature map of layer 2	0.010	0.074	0.071
Feature map of layer 4	0.003	0.034	0.032
Feature map of layer 6	0.002	0.029	0.027

*Note*: The accuracy is shown for using feature maps of second, fourth and sixth layers.

As described in the Section 1, when one-hot coding representation of four DNA nucleotides is used in the CNN, a filter with a one-dimensional convolution operation can be considered as a position weight matrix representing a motif (Alipanahi *et al.*, 2015; Aoki and Sakakibara, 2018; Zeng *et al.*, 2016). The position weight matrix is a generalization of the *k*-mer (word of length *k*). As our method using the style matrix of the *l*-th layer scans the DNA sequence with filters of the *l*-th layer and counts the matches with the position weight matrix represented by the filter, the style matrix can be considered as a generalization of the *k*-mers frequency.

More specifically, during pre-training of the feature-extraction CNN, sequence and structure motifs were extracted within filters that were important for taxonomic classification. The feature map is the filter responses for the input DNA sequence, and the genomic style is defined as the gram matrix of feature maps, thus representing the preference of those motifs. Filters in the feature-extraction CNN can be considered as position weight matrix representing motifs, and hence the gram matrix of feature maps can be considered as the preference distribution of probabilistic sequence motifs, including the probabilistic sequence signature (MetaProb), probabilistic *k*-mers statistics (MetaCon), empirical probabilistic distances of TNF (MetaBAT2), textual representations of sequence data (MrGBP) and the distribution of a carefully selected set of *k*-mers [MetaCluster (Yang *et al.*, 2010)]. Therefore, the genomic style can offer a unified framework for these variations in the *k*-mers frequency.

Most existing practical binning methods aimed to obtain highly reliable bins and hence excluded a large fraction of contigs as irrelevant. The binning accuracy of existing binning methods in the case of excluding unclassified contigs is shown in Supplementary Table S2. As our method has not implemented such function to detect and exclude irrelevant contigs, our method was not included in that performance evaluation. The implementation of such function of eliminating irrelevant contigs and obtaining reliable bins is one of our future studies. In addition, when performing agglomerative clustering, the true number of bacterial species (clusters) was given. Although this assumption is not practical, there exist several methods (Beghini *et al.*, 2021) for estimating the taxonomic decomposition and the number of species in the metagenome. Methods for determining the appropriate number of clusters automatically will be evaluated in our future studies.

## 6 Conclusions

In this study, we proposed a new concept named as ‘genomic style’, which is a feature of genomic sequences that are not limited to base composition and *k*-mers frequency, as well as a method for binning metagenomic sequences using the genomic style. We first constructed a CNN-based feature-extraction model for modeling the content of DNA sequence by performing species classification learning. The genomic style was extracted from the style matrix calculated using the trained feature-extraction CNN model, and binning of the metagenome sequence was performed using the style matrix. The binning results revealed that the genomic style can be a DNA sequence feature unique to the bacterial species for the accurate DNA sequence classification. We also investigated what kind of sequence features the style matrix acquired. It turned out that some basic sequence features, such as the GC content and DNA motifs were captured in the style matrix of the shallow layers.

A previous study (Simonyan and Zisserman, 2014) using the CNN for image recognition revealed that the deeper hidden layers captured higher-order features of the image. Further studies are needed to clarify the type of higher-order features of DNA sequence that can be captured by the style matrix in a deeper layer. In order to improve the accuracy of binning of the metagenome, it is also our future work to improve the feature-extraction CNN model to extract more accurate genomic styles specific to the bacterial species.

## Authors’ contributions

Y.Y. implemented the software, analyzed data and compared with the existing methods. A.H. implemented the software and analyzed data. Y.A. and M.A. analyzed data. Y.S. designed and supervised the research, analyzed data and wrote the article. All authors read and approved the final manuscript.

## Funding

This work was supported by JST, CREST Grant Number JPMJCR20S3, Japan. This work was also supported by AMED under Grant Number JP19gm6010006 and a Grant-in-Aid for Scientific Research on Innovative Areas ‘Frontier Research on Chemical Communications’ [no. 17H06410] from the Ministry of Education, Culture, Sports, Science and Technology of Japan.

*Conflict of Interest*: The authors declare that they have no competing interests.

## Supplementary Material

vbab039_Supplementary_Data
