# Supplementary material for: Genomic style: yet another deep-learning approach to characterize bacterial genome sequences
Source: Bioinform Adv. 2021 Dec 1;1(1):vbab039. doi: 10.1093/bioadv/vbab039 (PMC9710696; doi:10.1093/bioadv/vbab039)
Supplement: vbab039_Supplementary_Data [file vbab039_supplementary_data.pdf]

Supplementary Information:

"Genomic style: yet another deep-learning approach to characterize bacterial genome sequences"

Yuka Yoshimura, Akifumi Hamada, Yohann Augey, Manato Akiyama, and Yasubumi Sakakibara*

## Supplemental Sections

***Binning Evaluation Metrics: Homogeneity and Completeness***

A homogeneity score $h$ and a completeness score $c$ (Rosenberg et al., 2007) are based on entropy. Assume a data set consists of $n$ species: a set of species, $C = \{c_i | i = 1, \ldots, n\}$, and a set of clusters is obtained: $K = \{k_i | i = 1, \ldots, m\}$. $a_{ij}$ is the number of DNA sequences that belong to species $c_i$ and to cluster $k_i$. Then, the homogeneity score $h$ is defined as follows:

$$h = \begin{cases} 1 & \text{if } H(C, K) = 0 \\ 1 - \dfrac{H(C|K)}{H(C)} & \text{else} \end{cases}$$

where

$$H(C|K) = -\sum_{k=1}^{|K|} \sum_{c=1}^{|C|} \frac{a_{ck}}{N} \log \frac{a_{ck}}{\sum_{c=1}^{|C|} a_{ck}}$$

$$H(C) = -\sum_{c=1}^{|C|} \frac{\sum_{k=1}^{|K|} a_{ck}}{n} \log \frac{\sum_{k=1}^{|K|} a_{ck}}{n}$$

Also, the completeness score $c$ is defined as follows:

$$c = \begin{cases} 1 & \text{if } H(K, C) = 0 \\ 1 - \dfrac{H(K|C)}{H(K)} & \text{else} \end{cases}$$

where

$$H(K|C) = -\sum_{c=1}^{|C|} \sum_{k=1}^{|K|} \frac{a_{ck}}{N} \log \frac{a_{ck}}{\sum_{k=1}^{|K|} a_{ck}}$$

$$H(K) = -\sum_{k=1}^{|K|} \frac{\sum_{c=1}^{|C|} a_{ck}}{n} \log \frac{\sum_{c=1}^{|C|} a_{ck}}{n}$$

# Supplemental Tables and Figures

**Supplemental Table S1.** The 206 species used in the training and test datasets and their taxonomic hierarchy.

| phylum | class | order | family | genus | species |
|---|---|---|---|---|---|
| Actinobacteria | Actinobacteria | Corynebacteriales | Corynebacteriaceae | Corynebacterium | diphtheriae |
| Actinobacteria | Actinobacteria | Corynebacteriales | Mycobacteriaceae | Mycobacterium | leprae |
| Actinobacteria | Actinobacteria | Corynebacteriales | Nocardiaceae | Nocardia | brasiliensis |
| Actinobacteria | Actinobacteria | Corynebacteriales | Nocardiaceae | Nocardia | terpenica |
| Actinobacteria | Actinobacteria | Corynebacteriales | Nocardiaceae | Rhodococcus | biphenylivorans |
| Actinobacteria | Actinobacteria | Micromonosporales | Micromonosporaceae | Micromonospora | tulbaghiae |
| Actinobacteria | Actinobacteria | Micromonosporales | Micromonosporaceae | Plantactinospora | spbb1 |
| Actinobacteria | Actinobacteria | Micromonosporales | Micromonosporaceae | Plantactinospora | spbc1 |
| Actinobacteria | Actinobacteria | Micromonosporales | Micromonosporaceae | Plantactinospora | spkbs50 |
| Actinobacteria | Actinobacteria | Pseudonocardiales | Pseudonocardiaceae | Amycolatopsis | albispora |
| Actinobacteria | Actinobacteria | Streptomycetales | Streptomycetaceae | Kitasatospora | aureofaciens |
| Actinobacteria | Actinobacteria | Streptomycetales | Streptomycetaceae | Streptomyces | atratus |
| Actinobacteria | Actinobacteria | Streptomycetales | Streptomycetaceae | Streptomyces | dengpaensis |
| Actinobacteria | Actinobacteria | Streptomycetales | Streptomycetaceae | Streptomyces | fradiae |
| Actinobacteria | Actinobacteria | Streptomycetales | Streptomycetaceae | Streptomyces | koyangensis |
| Actinobacteria | Actinobacteria | Streptomycetales | Streptomycetaceae | Streptomyces | lunaelactis |
| Actinobacteria | Actinobacteria | Streptomycetales | Streptomycetaceae | Streptomyces | luteoverticillatus |
| Actinobacteria | Actinobacteria | Streptomycetales | Streptomycetaceae | Streptomyces | lydicus |
| Actinobacteria | Actinobacteria | Streptomycetales | Streptomycetaceae | Streptomyces | spongiicola |
| Actinobacteria | Actinobacteria | Streptomycetales | Streptomycetaceae | Streptomyces | tirandamycinicus |
| Bacteroidetes | Bacteroidia | Bacteroidales | Bacteroidaceae | Bacteroides | caccae |
| Bacteroidetes | Bacteroidia | Bacteroidales | Bacteroidaceae | Bacteroides | fragilis |
| Bacteroidetes | Bacteroidia | Bacteroidales | Bacteroidaceae | Bacteroides | ovatus |
| Bacteroidetes | Bacteroidia | Bacteroidales | Bacteroidaceae | Bacteroides | thetaiotaomicron |
| Bacteroidetes | Bacteroidia | Bacteroidales | Muribaculaceae | Duncaniella | spB8 |
| Bacteroidetes | Bacteroidia | Bacteroidales | Muribaculaceae | Muribaculum | intestinale |
| Bacteroidetes | Bacteroidia | Bacteroidales | Odoribacteraceae | Odoribacter | splanchnicus |
| Bacteroidetes | Bacteroidia | Bacteroidales | Porphyromonadaceae | Porphyromonas | gingivalis |
| Bacteroidetes | Bacteroidia | Bacteroidales | Prevotellaceae | Prevotella | melaninogenica |
| Bacteroidetes | Bacteroidia | Bacteroidales | Rikenellaceae | Alistipes | finegoldii |
| Bacteroidetes | Bacteroidia | Marinilabiliales | Prolixibacteraceae | Draconibacterium | orientale |
| Cyanobacteria | Oscillatoriophycideae | Oscillatoriales | Oscillatoriaceae | Moorea | producens |
| Firmicutes | Bacilli | Bacillales | Bacillaceae | Bacillus | amyloliquefaciens |
| Firmicutes | Bacilli | Bacillales | Bacillaceae | Bacillus | anthracis |
| Firmicutes | Bacilli | Bacillales | Bacillaceae | Bacillus | beveridgei |
| Firmicutes | Bacilli | Bacillales | Bacillaceae | Bacillus | muralis |
| Firmicutes | Bacilli | Bacillales | Bacillaceae | Bacillus | mycoides |
| Firmicutes | Bacilli | Bacillales | Bacillaceae | Bacillus | oceanisediminis |
| Firmicutes | Bacilli | Bacillales | Bacillaceae | Bacillus | sp.IHB |
| Firmicutes | Bacilli | Bacillales | Bacillaceae | Bacillus | subtilis |
| Firmicutes | Bacilli | Bacillales | Bacillaceae | Bacillus | thuringensis |
| Firmicutes | Bacilli | Bacillales | Bacillaceae | Bacillus | velezensis |
| Firmicutes | Bacilli | Bacillales | Bacillaceae | Geobacillus | thermoleovorans |
| Firmicutes | Bacilli | Bacillales | Listeriaceae | Listeria | monocytogenes |
| Firmicutes | Bacilli | Bacillales | Paenibacillaceae | Brevibacillus | brevis |
| Firmicutes | Bacilli | Bacillales | Paenibacillaceae | Brevibacillus | laterosporus |
| Firmicutes | Bacilli | Bacillales | Staphylococcaceae | Staphylococcus | argenteus |
| Firmicutes | Bacilli | Bacillales | Staphylococcaceae | Staphylococcus | aureus |
| Firmicutes | Bacilli | Bacillales | Staphylococcaceae | Staphylococcus | epidermidis |
| Firmicutes | Bacilli | Lactobacillales | Enterococcaceae | Enterococcus | faecalis |
| Firmicutes | Bacilli | Lactobacillales | Enterococcaceae | Enterococcus | faecium |
| Firmicutes | Bacilli | Lactobacillales | Lactobacillaceae | Lactobacillus | casei |
| Firmicutes | Bacilli | Lactobacillales | Lactobacillaceae | Lactobacillus | curvatus |
| Firmicutes | Bacilli | Lactobacillales | Lactobacillaceae | Lactobacillus | delbrueckii |
| Firmicutes | Bacilli | Lactobacillales | Lactobacillaceae | Lactobacillus | fermentum |
| Firmicutes | Bacilli | Lactobacillales | Lactobacillaceae | Lactobacillus | lindneri |
| Firmicutes | Bacilli | Lactobacillales | Lactobacillaceae | Lactobacillus | paracasei |

| | | | | | |
|---|---|---|---|---|---|
| Firmicutes | Bacilli | Lactobacillales | Lactobacillaceae | Lactobacillus | plantarum |
| Firmicutes | Bacilli | Lactobacillales | Lactobacillaceae | Lactobacillus | rhamnosus |
| Firmicutes | Bacilli | Lactobacillales | Lactobacillaceae | Lactobacillus | salivarius |
| Firmicutes | Bacilli | Lactobacillales | Lactobacillaceae | Lactobacillus | sanfranciscensis |
| Firmicutes | Bacilli | Lactobacillales | Streptococcaceae | Streptococcus | agalactiae |
| Firmicutes | Bacilli | Lactobacillales | Streptococcaceae | Streptococcus | cristatus |
| Firmicutes | Bacilli | Lactobacillales | Streptococcaceae | Streptococcus | equi |
| Firmicutes | Bacilli | Lactobacillales | Streptococcaceae | Streptococcus | pneumoniae |
| Firmicutes | Bacilli | Lactobacillales | Streptococcaceae | Streptococcus | pyogenes |
| Firmicutes | Bacilli | Lactobacillales | Streptococcaceae | Streptococcus | salivarius |
| Firmicutes | Bacilli | Lactobacillales | Streptococcaceae | Streptococcus | sp.A12 |
| Firmicutes | Bacilli | Lactobacillales | Streptococcaceae | Streptococcus | suis |
| Firmicutes | Clostridia | Clostridiales | Clostridiaceae | Clostridium | spawrp |
| Proteobacteria | Alphaproteobacteria | Magnetococcales | Magnetococcaceae | Magnetococcus | marinus |
| Proteobacteria | Alphaproteobacteria | Rhizobiales | Bradyrhizobiaceae | Bradyrhizobium | diazoefficiens |
| Proteobacteria | Alphaproteobacteria | Rhizobiales | Brucellaceae | Brucella | abortus |
| Proteobacteria | Alphaproteobacteria | Rhizobiales | Brucellaceae | Brucella | canis |
| Proteobacteria | Alphaproteobacteria | Rhizobiales | Brucellaceae | Brucella | ceti |
| Proteobacteria | Alphaproteobacteria | Rhizobiales | Brucellaceae | Brucella | melitensis |
| Proteobacteria | Alphaproteobacteria | Rhizobiales | Brucellaceae | Brucella | suis |
| Proteobacteria | Alphaproteobacteria | Rhizobiales | Methylobacteriaceae | Methylobacterium | extorquens |
| Proteobacteria | Alphaproteobacteria | Rhizobiales | Methylobacteriaceae | Methylobacterium | populi |
| Proteobacteria | Alphaproteobacteria | Rhizobiales | Phyllobacteriaceae | Aminobacter | aminovorans |
| Proteobacteria | Alphaproteobacteria | Rhizobiales | Phyllobacteriaceae | Mesorhizobium | ciceri |
| Proteobacteria | Alphaproteobacteria | Rhizobiales | Phyllobacteriaceae | Mesorhizobium | loti |
| Proteobacteria | Alphaproteobacteria | Rhizobiales | Phyllobacteriaceae | Mesorhizobium | oceanicum |
| Proteobacteria | Alphaproteobacteria | Rhizobiales | Phyllobacteriaceae | Mesorhizobium | spm1d |
| Proteobacteria | Alphaproteobacteria | Rhizobiales | Phyllobacteriaceae | Mesorhizobium | spm2a |
| Proteobacteria | Alphaproteobacteria | Rhizobiales | Phyllobacteriaceae | Mesorhizobium | spm3a |
| Proteobacteria | Alphaproteobacteria | Rhizobiales | Rhizobiaceae | Ensifer | sojae |
| Proteobacteria | Alphaproteobacteria | Rhizobiales | Rhizobiaceae | Sinorhizobium | fredii |
| Proteobacteria | Alphaproteobacteria | Rhizobiales | Xanthobacteraceae | Azorhizobium | caulinodans |
| Proteobacteria | Alphaproteobacteria | Rhodobacterales | Rhodobacteraceae | Epibacterium | mobile |
| Proteobacteria | Alphaproteobacteria | Rhodobacterales | Rhodobacteraceae | Paracoccus | mutanolyticus |
| Proteobacteria | Alphaproteobacteria | Rhodobacterales | Rhodobacteraceae | Phaeobacter | inhibens |
| Proteobacteria | Alphaproteobacteria | Rhodobacterales | Rhodobacteraceae | Rhodobacter | sphaeroides |
| Proteobacteria | Alphaproteobacteria | Rickettsiales | Anaplasmataceae | Anaplasma | phagocytophilum |
| Proteobacteria | Alphaproteobacteria | Rickettsiales | Anaplasmataceae | Ehrlichia | ruminantium |
| Proteobacteria | Alphaproteobacteria | Rickettsiales | Rickettsiaceae | Rickettsia | japonica |
| Proteobacteria | Alphaproteobacteria | Rickettsiales | Rickettsiaceae | Rickettsia | prowazekii |
| Proteobacteria | Alphaproteobacteria | Sphingomonadales | Erythrobacteraceae | Altererythrobacter | dongtanensis |
| Proteobacteria | Alphaproteobacteria | Sphingomonadales | Erythrobacteraceae | Altererythrobacter | ishigakiensis |
| Proteobacteria | Alphaproteobacteria | Sphingomonadales | Erythrobacteraceae | Croceicoccus | naphthovorans |
| Proteobacteria | Alphaproteobacteria | Sphingomonadales | Erythrobacteraceae | Erythrobacter | atlanticus |
| Proteobacteria | Alphaproteobacteria | Sphingomonadales | Erythrobacteraceae | Erythrobacter | litoralis |
| Proteobacteria | Alphaproteobacteria | Sphingomonadales | Sphingomonadaceae | Novosphingobium | pentaromativorans |
| Proteobacteria | Alphaproteobacteria | Sphingomonadales | Sphingomonadaceae | Novosphingobium | resinovorum |
| Proteobacteria | Alphaproteobacteria | Sphingomonadales | Sphingomonadaceae | Novosphingobium | sp.PP1Y |
| Proteobacteria | Alphaproteobacteria | Sphingomonadales | Sphingomonadaceae | Sphingobium | japonicum |
| Proteobacteria | Alphaproteobacteria | Sphingomonadales | Sphingomonadaceae | Sphingobium | sp.RAC03 |
| Proteobacteria | Alphaproteobacteria | Sphingomonadales | Sphingomonadaceae | Sphingobium | sp.SYK-6 |
| Proteobacteria | Alphaproteobacteria | Sphingomonadales | Sphingomonadaceae | Sphingobium | sp.TKS |
| Proteobacteria | Alphaproteobacteria | Sphingomonadales | Sphingomonadaceae | Sphingobium | sp.YBL2 |
| Proteobacteria | Betaproteobacteria | Burkholderiales | Alcaligenaceae | Achromobacter | xylosoxidans |
| Proteobacteria | Betaproteobacteria | Burkholderiales | Alcaligenaceae | Advenella | kashmirensis |
| Proteobacteria | Betaproteobacteria | Burkholderiales | Alcaligenaceae | Bordetella | bronchiseptica |
| Proteobacteria | Betaproteobacteria | Burkholderiales | Alcaligenaceae | Bordetella | holmesii |
| Proteobacteria | Betaproteobacteria | Burkholderiales | Alcaligenaceae | Bordetella | pertussis |
| Proteobacteria | Betaproteobacteria | Burkholderiales | Alcaligenaceae | Castellaniella | defragrans |
| Proteobacteria | Betaproteobacteria | Burkholderiales | Alcaligenaceae | Pusillimonas | sp.T7-7 |
| Proteobacteria | Betaproteobacteria | Burkholderiales | Burkholderiaceae | Burkholderia | cepacia |
| Proteobacteria | Betaproteobacteria | Burkholderiales | Burkholderiaceae | Burkholderia | pseudomallei |
| Proteobacteria | Betaproteobacteria | Burkholderiales | Burkholderiaceae | Burkholderia | ubonensis |
| Proteobacteria | Betaproteobacteria | Burkholderiales | Burkholderiaceae | Burkholderia | vietnamiensis |
| Proteobacteria | Betaproteobacteria | Burkholderiales | Burkholderiaceae | Ralstonia | solanacearum |
| Proteobacteria | Betaproteobacteria | Burkholderiales | Comamonadaceae | Comamonas | thiooxydans |
| Proteobacteria | Betaproteobacteria | Burkholderiales | Comamonadaceae | Serpentinomonas | mccroryi |
| Proteobacteria | Betaproteobacteria | Burkholderiales | Oxalobacteraceae | Massilia | umbonata |
| Proteobacteria | Betaproteobacteria | Neisseriales | Neisseriaceae | Neisseria | elongata |
| Proteobacteria | Betaproteobacteria | Neisseriales | Neisseriaceae | Neisseria | gonorrhoeae |
| Proteobacteria | Betaproteobacteria | Neisseriales | Neisseriaceae | Neisseria | meningitidis |

| | | | | | |
|---|---|---|---|---|---|
| Proteobacteria | Betaproteobacteria | Neisseriales | Neisseriaceae | Neisseria | sicca |
| Proteobacteria | Betaproteobacteria | Neisseriales | Neisseriaceae | Neisseria | subflava |
| Proteobacteria | Betaproteobacteria | Nitrosomonadales | Gallionellaceae | Sulfuricella | denitrificans |
| Proteobacteria | Betaproteobacteria | Nitrosomonadales | Sterolibacteriaceae | Sulfuritalea | hydrogenivorans |
| Proteobacteria | Deltaproteobacteria | Bradymonadales | Bradymonadaceae | Bradymonas | sediminis |
| Proteobacteria | Deltaproteobacteria | Desulfobacterales | Desulfobacteraceae | Desulfatibacillum | aliphaticivorans |
| Proteobacteria | Deltaproteobacteria | Desulfobacterales | Desulfobacteraceae | Desulfococcus | multivorans |
| Proteobacteria | Deltaproteobacteria | Desulfovibrionales | Desulfovibrionaceae | Desulfovibrio | carbinolicus |
| Proteobacteria | Deltaproteobacteria | Desulfovibrionales | Desulfovibrionaceae | Desulfovibrio | desulfuricans |
| Proteobacteria | Deltaproteobacteria | Desulfovibrionales | Desulfovibrionaceae | Desulfovibrio | ferrophilus |
| Proteobacteria | Deltaproteobacteria | Desulfovibrionales | Desulfovibrionaceae | Lawsonia | intracellularis |
| Proteobacteria | Deltaproteobacteria | Desulfuromonadales | Geobacteraceae | Geoalkalibacter | subterraneus |
| Proteobacteria | Deltaproteobacteria | Desulfuromonadales | Geobacteraceae | Geobacter | sulfurreducens |
| Proteobacteria | Deltaproteobacteria | Syntrophobacterales | Syntrophobacteraceae | Desulfoglaeba | alkanexedens |
| Proteobacteria | Epsilonproteobacteria | Campylobacterales | Campylobacteraceae | Arcobacter | butzleri |
| Proteobacteria | Epsilonproteobacteria | Campylobacterales | Campylobacteraceae | Campylobacter | coli |
| Proteobacteria | Epsilonproteobacteria | Campylobacterales | Campylobacteraceae | Campylobacter | jejuni |
| Proteobacteria | Epsilonproteobacteria | Campylobacterales | Helicobacteraceae | Helicobacter | cetorum |
| Proteobacteria | Epsilonproteobacteria | Campylobacterales | Helicobacteraceae | Helicobacter | pylori |
| Proteobacteria | Epsilonproteobacteria | Campylobacterales | Helicobacteraceae | Sulfuricurvum | candidatussulfuricurvum |
| Proteobacteria | Gammaproteobacteria | Alteromonadales | Alteromonadaceae | Alteromonas | macleodii |
| Proteobacteria | Gammaproteobacteria | Alteromonadales | Alteromonadaceae | Alteromonas | mediterranea |
| Proteobacteria | Gammaproteobacteria | Alteromonadales | Shewanellaceae | Shewanella | baltica |
| Proteobacteria | Gammaproteobacteria | Alteromonadales | Shewanellaceae | Shewanella | pealeana |
| Proteobacteria | Gammaproteobacteria | Alteromonadales | Shewanellaceae | Shewanella | sediminis |
| Proteobacteria | Gammaproteobacteria | Alteromonadales | Shewanellaceae | Shewanella | sp.ANA-3 |
| Proteobacteria | Gammaproteobacteria | Alteromonadales | Shewanellaceae | Shewanella | sp.MR-4 |
| Proteobacteria | Gammaproteobacteria | Enterobacterales | Enterobacteriaceae | Citrobacter | amalonaticus |
| Proteobacteria | Gammaproteobacteria | Enterobacterales | Enterobacteriaceae | Citrobacter | freundii |
| Proteobacteria | Gammaproteobacteria | Enterobacterales | Enterobacteriaceae | Citrobacter | koseri |
| Proteobacteria | Gammaproteobacteria | Enterobacterales | Enterobacteriaceae | Citrobacter | rodentium |
| Proteobacteria | Gammaproteobacteria | Enterobacterales | Enterobacteriaceae | Cronobacter | sakazakii |
| Proteobacteria | Gammaproteobacteria | Enterobacterales | Enterobacteriaceae | Enterobacter | aerogenes |
| Proteobacteria | Gammaproteobacteria | Enterobacterales | Enterobacteriaceae | Enterobacter | cloacae |
| Proteobacteria | Gammaproteobacteria | Enterobacterales | Enterobacteriaceae | Enterobacter | hormaechei |
| Proteobacteria | Gammaproteobacteria | Enterobacterales | Enterobacteriaceae | Enterobacter | soli |
| Proteobacteria | Gammaproteobacteria | Enterobacterales | Enterobacteriaceae | Enterobacter | sp.FY-07 |
| Proteobacteria | Gammaproteobacteria | Enterobacterales | Enterobacteriaceae | Enterobacter | sp.ODB01 |
| Proteobacteria | Gammaproteobacteria | Enterobacterales | Enterobacteriaceae | Enterobacter | spEA1 |
| Proteobacteria | Gammaproteobacteria | Enterobacterales | Enterobacteriaceae | Erwinia | amylovora |
| Proteobacteria | Gammaproteobacteria | Enterobacterales | Enterobacteriaceae | Erwinia | pyrifoliae |
| Proteobacteria | Gammaproteobacteria | Enterobacterales | Enterobacteriaceae | Erwinia | sp.EM595 |
| Proteobacteria | Gammaproteobacteria | Enterobacterales | Enterobacteriaceae | Erwinia | tasmaniensis |
| Proteobacteria | Gammaproteobacteria | Enterobacterales | Enterobacteriaceae | Escherichia | albertii |
| Proteobacteria | Gammaproteobacteria | Enterobacterales | Enterobacteriaceae | Escherichia | coli |
| Proteobacteria | Gammaproteobacteria | Enterobacterales | Enterobacteriaceae | Escherichia | fergusonii |
| Proteobacteria | Gammaproteobacteria | Enterobacterales | Enterobacteriaceae | Klebsiella | pneumoniae |
| Proteobacteria | Gammaproteobacteria | Enterobacterales | Enterobacteriaceae | Klebsiella | variicola |
| Proteobacteria | Gammaproteobacteria | Enterobacterales | Enterobacteriaceae | Salmonella | enterica |
| Proteobacteria | Gammaproteobacteria | Enterobacterales | Enterobacteriaceae | Shigella | dysenteriae |
| Proteobacteria | Gammaproteobacteria | Enterobacterales | Enterobacteriaceae | Shigella | flexneri |
| Proteobacteria | Gammaproteobacteria | Enterobacterales | Enterobacteriaceae | Shigella | sonnei |
| Proteobacteria | Gammaproteobacteria | Enterobacterales | Enterobacteriaceae | Shigella | sp.PAMC |
| Proteobacteria | Gammaproteobacteria | Enterobacterales | Morganellaceae | Photorhabdus | laumondii |
| Proteobacteria | Gammaproteobacteria | Enterobacterales | Pectobacteriaceae | Pectobacterium | atrosepticum |
| Proteobacteria | Gammaproteobacteria | Enterobacterales | Pectobacteriaceae | Pectobacterium | parmentieri |
| Proteobacteria | Gammaproteobacteria | Enterobacterales | Yersiniaceae | Serratia | marcescens |
| Proteobacteria | Gammaproteobacteria | Enterobacterales | Yersiniaceae | Serratia | plymuthica |
| Proteobacteria | Gammaproteobacteria | Enterobacterales | Yersiniaceae | Yersinia | enterocolitica |
| Proteobacteria | Gammaproteobacteria | Enterobacterales | Yersiniaceae | Yersinia | frederiksenii |
| Proteobacteria | Gammaproteobacteria | Enterobacterales | Yersiniaceae | Yersinia | pestis |
| Proteobacteria | Gammaproteobacteria | Enterobacterales | Yersiniaceae | Yersinia | pseudotuberculosis |
| Proteobacteria | Gammaproteobacteria | Immundisolibacterales | Immundisolibacteraceae | Immundisolibacter | cernigliae |
| Proteobacteria | Gammaproteobacteria | Legionellales | Legionellaceae | Legionella | pneumophila |
| Proteobacteria | Gammaproteobacteria | Pasteurellales | Pasteurellaceae | Bibersteinia | trehalosi |
| Proteobacteria | Gammaproteobacteria | Pasteurellales | Pasteurellaceae | Haemophilus | influenzae |
| Proteobacteria | Gammaproteobacteria | Pseudomonadales | Moraxellaceae | Acinetobacter | baumannii |
| Proteobacteria | Gammaproteobacteria | Pseudomonadales | Pseudomonadaceae | Pseudomonas | avellanae |
| Proteobacteria | Gammaproteobacteria | Pseudomonadales | Pseudomonadaceae | Pseudomonas | entomophila |
| Proteobacteria | Gammaproteobacteria | Pseudomonadales | Pseudomonadaceae | Pseudomonas | fulva |
| Proteobacteria | Gammaproteobacteria | Pseudomonadales | Pseudomonadaceae | Pseudomonas | monteilii |

| | | | | | |
|---|---|---|---|---|---|
| Proteobacteria | Gammaproteobacteria | Pseudomonadales | Pseudomonadaceae | Pseudomonas | syringae |
| Proteobacteria | Gammaproteobacteria | Vibrionales | Vibrionaceae | Vibrio | anguillarum |
| Proteobacteria | Gammaproteobacteria | Vibrionales | Vibrionaceae | Vibrio | cholerae |
| Proteobacteria | Gammaproteobacteria | Vibrionales | Vibrionaceae | Vibrio | parahaemolyticus |
| Proteobacteria | Gammaproteobacteria | Xanthomonadales | Xanthomonadaceae | Xanthomonas | campestris |
| Proteobacteria | Gammaproteobacteria | Xanthomonadales | Xanthomonadaceae | Xanthomonas | oryzae |
| Proteobacteria | Gammaproteobacteria | Xanthomonadales | Xanthomonadaceae | Xanthomonas | vasicola |

**Supplemental Table S2.** Binning accuracy of existing binning methods in the case of excluding unclassified contigs (on CAMI challenge dataset).

| | MetaBAT2 | CONCOCT | MaxBin2 | MrGBP |
|---|---|---|---|---|
| ARI | 0.941 | 0.828 | 0.740 | 0.249 |
| Homogeneity | 0.915 | 0.854 | 0.791 | 0.298 |
| Completeness | 0.942 | 0.838 | 0.749 | 0.942 |
| Num. of bins | 35 | 46 | 45 | 9 |
| Num. of unclassified | 15,592 | 11,063 | 11,182 | 11,091 |

**Supplemental Figure S1.** The taxonomic hierarchy diagram for 206 species used in the training and test datasets.

| phylum | class | order | family | genus | species |
|---|---|---|---|---|---|
| Actinobacteria | Actinobacteria | Corynebacteriales | Corynebacteriaceae | Corynebacterium | diphtheriae |
| | | | Mycobacteriaceae | Mycobacterium | leprae |
| | | | Nocardiaceae | Nocardia | brasiliensis |
| | | | | | terpenica |
| | | | | Rhodococcus | biphenylivorans |
| | | Micromonosporales | Micromonosporaceae | Micromonospora | tulbaghiae |
| | | | | Plantactinospora | spbI |
| | | | | | spbcl |
| | | | | | spkbs50 |
| | | Pseudonocardiales | Pseudonocardiaceae | Amycolatopsis | albispora |
| | | Streptomycetales | Streptomycetaceae | Kitasatospora | aureofaciens |
| | | | | Streptomyces | atratus |
| | | | | | dengpaensis |
| | | | | | fradiae |
| | | | | | koyangensis |
| | | | | | lunaelactis |
| | | | | | luteoverticillatus |
| | | | | | lydicus |
| | | | | | spongicola |
| | | | | | tirandamycinicus |
| Bacteroidetes | Bacteroidia | Bacteroidales | Bacteroidaceae | Bacteroides | caccae |
| | | | | | fragilis |
| | | | | | ovatus |
| | | | | | thetaiotaomicron |
| | | | Muribaculaceae | Duncaniella | spB8 |
| | | | | Muribaculum | intestinale |
| | | | Odoribacteraceae | Odoribacter | splanchnicus |
| | | | Porphyromonadaceae | Porphyromonas | gingivalis |
| | | | Prevotellaceae | Prevotella | melaninogenica |
| | | | Rikenellaceae | Alistipes | finegoldii |
| | | Marinilabiliales | Prolixibacteraceae | Draconibacterium | orientale |
| Cyanobacteria | Oscillatoriophycideae | Oscillatoriales | Oscillatoriaceae | Moorea | producens |
| Firmicutes | Bacilli | Bacillales | Bacillaceae | Bacillus | amyloliquefaciens |
| | | | | | anthracis |
| | | | | | beveridgei |
| | | | | | muralis |
| | | | | | mycoides |
| | | | | | oceanisediminis |
| | | | | | sp.IHB |
| | | | | | subtilis |
| | | | | | thuringiensis |
| | | | | | velezensis |
| | | | | Geobacillus | thermoleovorans |
| | | | Listeriaceae | Listeria | monocytogenes |
| | | | Paenibacillaceae | Brevibacillus | brevis |
| | | | | | laterosporus |
| | | | Staphylococcaceae | Staphylococcus | argenteus |
| | | | | | aureus |
| | | | | | epidermidis |
| | | Lactobacillales | Enterococcaceae | Enterococcus | faecalis |
| | | | | | faecium |
| | | | Lactobacillaceae | Lactobacillus | casei |
| | | | | | curvatus |
| | | | | | delbrueckii |
| | | | | | fermentum |
| | | | | | lindneri |
| | | | | | paracasei |
| | | | | | plantarum |
| | | | | | rhamnosus |
| | | | | | salivarius |
| | | | | | sanfranciscensis |
| | | | Streptococcaceae | Streptococcus | agalactiae |
| | | | | | cristatus |
| | | | | | equi |
| | | | | | pneumoniae |
| | | | | | pyogenes |
| | | | | | salivarius |
| | | | | | sp.A12 |
| | | | | | suis |
| | Clostridia | Clostridiales | Clostridiaceae | Clostridium | spawp |
| Proteobacteria | Alphaproteobacteria | Magnetococcales | Magnetococcaceae | Magnetococcus | marinus |
| | | Rhizobiales | Bradyrhizobiaceae | Bradyrhizobium | diazoefficiens |
| | | | Brucellaceae | Brucella | abortus |
| | | | | | canis |
| | | | | | ceti |
| | | | | | melitensis |
| | | | | | suis |
| | | | Methylobacteriaceae | Methylobacterium | extorquens |
| | | | | | populi |
| | | | Phyllobacteriaceae | Aminobacter | aminovorans |
| | | | | Mesorhizobium | ciceri |
| | | | | | loti |
| | | | | | oceanicum |
| | | | | | spm1d |
| | | | | | spm2a |
| | | | | | spm3a |
| | | | Rhizobiaceae | Ensifer | sojae |
| | | | | Sinorhizobium | fredii |
| | | | Xanthobacteraceae | Azorhizobium | caulinodans |
| | | Rhodobacterales | Rhodobacteraceae | Epibacterium | mobile |
| | | | | Paracoccus | mutanolyticus |
| | | | | Phaeobacter | inhibens |
| | | | | Rhodobacter | sphaeroides |
| | | Rickettsiales | Anaplasmataceae | Anaplasma | phagocytophilum |
| | | | | Ehrlichia | ruminantium |
| | | | Rickettsiaceae | Rickettsia | japonica |
| | | | | | prowazekii |
| | | Sphingomonadales | Erythrobacteraceae | Altererythrobacter | dongtanensis |
| | | | | | ishigakiensis |
| | | | | Croceicoccus | naphthovorans |
| | | | | Erythrobacter | atlanticus |
| | | | | | litoralis |
| | | | Sphingomonadaceae | Novosphingobium | pentaromativorans |
| | | | | | resinovorum |
| | | | | | sp.PP1Y |
| | | | | Sphingobium | japonicum |
| | | | | | sp.RAC03 |
| | | | | | sp.SYK-6 |
| | | | | | sp.TKS |
| | | | | | sp.YBL2 |
| | Betaproteobacteria | Burkholderiales | Alcaligenaceae | Achromobacter | xylosoxidans |
| | | | | Advenella | kashmirensis |
| | | | | Bordetella | bronchiseptica |
| | | | | | holmesii |
| | | | | | pertussis |
| | | | | Castellaniella | defragrans |
| | | | | Pusillimonas | sp.T7-7 |
| | | | Burkholderiaceae | Burkholderia | cepacia |
| | | | | | pseudomallei |
| | | | | | ubonensis |
| | | | | | vietnamiensis |
| | | | | Ralstonia | solanacearum |
| | | | Comamonadaceae | Comamonas | thiooxydans |
| | | | | Serpentinomonas | mccroryi |
| | | | Oxalobacteraceae | Massilia | umbonata |
| | | Neisseriales | Neisseriaceae | Neisseria | elongata |
| | | | | | gonorrhoeae |
| | | | | | meningitidis |
| | | | | | sicca |
| | | | | | subflava |
| | | Nitrosomonadales | Gallionellaceae | Sulfuricella | denitrificans |
| | | | Sterolibacteriaceae | Sulfuritalea | hydrogenivorans |
| | Deltaproteobacteria | Bradymonadales | Bradymonadaceae | Bradymonas | sediminis |
| | | Desulfobacterales | Desulfobacteraceae | Desulfatibacillum | aliphaticivorans |
| | | | | Desulfococcus | multivorans |
| | | Desulfovibrionales | Desulfovibrionaceae | Desulfovibrio | carbinolicus |
| | | | | | desulfuricans |
| | | | | | ferrophilus |
| | | | | Lawsonia | intracellularis |
| | | Desulfuromonadales | Geobacteraceae | Geoalkalibacter | subterraneus |
| | | | | Geobacter | sulfurreducens |
| | | Syntrophobacterales | Syntrophobacteraceae | Desulfoglaeba | alkanexedens |
| | Epsilonproteobacteria | Campylobacterales | Campylobacteraceae | Arcobacter | butzleri |
| | | | | Campylobacter | coli |
| | | | | | jejuni |
| | | | Helicobacteraceae | Helicobacter | cetorum |
| | | | | | pylori |
| | | | | Sulfuricurvum | candidatussulfuricurvum |
| | Gammaproteobacteria | Alteromonadales | Alteromonadaceae | Alteromonas | macleodii |
| | | | | | mediterranea |
| | | | Shewanellaceae | Shewanella | baltica |
| | | | | | pealeana |
| | | | | | sediminis |
| | | | | | sp.ANA-3 |
| | | | | | sp.MR-4 |
| | | Enterobacterales | Enterobacteriaceae | Citrobacter | amalonaticus |
| | | | | | freundii |
| | | | | | koseri |
| | | | | | rodentium |
| | | | | Cronobacter | sakazakii |
| | | | | Enterobacter | aerogenes |
| | | | | | cloacae |
| | | | | | hormaechei |
| | | | | | soli |
| | | | | | sp.FY-07 |
| | | | | | sp.ODB01 |
| | | | | | spEA1 |
| | | | | Erwinia | amylovora |
| | | | | | pyrifoliae |
| | | | | | sp.EM595 |
| | | | | | tasmaniensis |
| | | | | Escherichia | albertii |
| | | | | | coli |
| | | | | | fergusonii |
| | | | | Klebsiella | pneumoniae |
| | | | | | variicola |
| | | | | Salmonella | enterica |
| | | | | Shigella | dysenteriae |
| | | | | | flexneri |
| | | | | | sonnei |
| | | | | | sp.PAMC |
| | | | Morganellaceae | Photorhabdus | laumondii |
| | | | Pectobacteriaceae | Pectobacterium | atrosepticum |
| | | | | | pamentieri |
| | | | Yersiniaceae | Serratia | marcescens |
| | | | | | plymuthica |
| | | | | Yersinia | enterocolitica |
| | | | | | frederiksenii |
| | | | | | pestis |
| | | | | | pseudotuberculosis |
| | | Immundisolibacterales | Immundisolibacteraceae | Immundisolibacter | cernigliae |
| | | Legionellales | Legionellaceae | Legionella | pneumophila |
| | | Pasteurellales | Pasteurellaceae | Bibersteinia | trehalosi |
| | | | | Haemophilus | influenzae |
| | | Pseudomonadales | Moraxellaceae | Acinetobacter | baumannii |
| | | | Pseudomonadaceae | Pseudomonas | avellanae |
| | | | | | entomophila |
| | | | | | fulva |
| | | | | | montelii |
| | | | | | syringae |
| | | Vibrionales | Vibrionaceae | Vibrio | anguillarum |
| | | | | | cholerae |
| | | | | | parahaemolyticus |
| | | Xanthomonadales | Xanthomonadaceae | Xanthomonas | campestris |
| | | | | | oryzae |
| | | | | | vasicola |

**Supplemental Figure S2.** The taxonomic hierarchy diagram for 67 species used in the test dataset.

| phylum | class | order | family | genus | species |
|---|---|---|---|---|---|
| Firmicutes | Bacilli | Bacillales | Bacillaceae | Bacillus | amyloliquefaciens |
| | | | | | anthracis |
| | | | | | beveridgei |
| | | | | | muralis |
| | | | | | oceanisediminis |
| | | | | | sp.IHB |
| | | | | | velezensis |
| | | | Paenibacillaceae | Brevibacillus | brevis |
| | | | | | laterosporus |
| | | Lactobacillales | Lactobacillaceae | Lactobacillus | casei |
| | | | | | curvatus |
| | | | | | delbrueckii |
| | | | | | lindneri |
| | | | | | paracasei |
| | | | | | rhamnosus |
| | | | | | salivarius |
| | | | | | sanfranciscensis |
| | | | Streptococcaceae | Streptococcus | cristatus |
| | | | | | salivarius |
| | | | | | sp.A12 |
| Proteobacteria | Alphaproteobacteria | Rhizobiales | Brucellaceae | Brucella | abortus |
| | | | | | canis |
| | | | | | ceti |
| | | | | | melitensis |
| | | | | | suis |
| | | | Methylobacteriaceae | Methylobacterium | extorquens |
| | | | | | populi |
| | | | Phyllobacteriaceae | Aminobacter | aminovorans |
| | | | | Mesorhizobium | ciceri |
| | | | | | oceanicum |
| | | | Xanthobacteraceae | Azorhizobium | caulinodans |
| | | Sphingomonadales | Erythrobacteraceae | Altererythrobacter | dongtanensis |
| | | | | | ishigakiensis |
| | | | | Croceicoccus | naphthovorans |
| | | | | Erythrobacter | atlanticus |
| | | | Sphingomonadaceae | Novosphingobium | pentaromativorans |
| | | | | | resinovorum |
| | | | | | sp.PP1Y |
| | | | | Sphingobium | japonicum |
| | | | | | sp.RAC03 |
| | | | | | sp.SYK-6 |
| | | | | | sp.TKS |
| | | | | | sp.YBL2 |
| | Betaprote -obacteria | Burkholderiales | Castellaniella | Castellaniella | defragrans |
| | | | | Pusillimonas | sp.T7-7 |
| | | | Burkholderiaceae | Burkholderiaceae | ubonensis |
| | | | | | vietnamiensis |
| | | Neisseriales | Neisseriaceae | Neisseria | elongata |
| | Gammaproteobacteria | Alteromonadales | Shewanellaceae | Shewanellaceae | pealeana |
| | | | | | sediminis |
| | | | | | sp.ANA-3 |
| | | | | | sp.MR-4 |
| | | Enterobacterales | Enterobacteriaceae | Citrobacter | amalonaticus |
| | | | | | freundii |
| | | | | | koseri |
| | | | | Enterobacter | aerogenes |
| | | | | | sp.FY-07 |
| | | | | | sp.ODB01 |
| | | | | Erwinia | amylovora |
| | | | | | pyrifoliae |
| | | | | | sp.EM595 |
| | | | | | tasmaniensis |
| | | | | Klebsiella | variicola |
| | | | | Shigella | dysenteriae |
| | | | | | sp.PAMC |
| | | | Yersiniaceae | Yersinia | frederiksenii |
| | | | | | pseudotuberculosis |

## References

Rosenberg, Andrew, and Julia Hirschberg. (2007). V-measure: A conditional entropy-based external cluster evaluation measure. *Proceedings of the 2007 joint conference on empirical methods in natural language processing and computational natural language learning (EMNLP-CoNLL)*.
